# The origin and widespread occurrence of *Sli*-based self-compatibility in potato

**DOI:** 10.1007/s00122-020-03627-8

**Published:** 2020-06-08

**Authors:** Corentin R. Clot, Clara Polzer, Charlotte Prodhomme, Cees Schuit, Christel J. M. Engelen, Ronald C. B. Hutten, Herman J. van Eck

**Affiliations:** 1 Plant Breeding, Wageningen University, P.O. Box 386, 6700 AJ Wageningen, The Netherlands; 2 Bejo Zaden B.V., Trambaan 1, 1749 CZ Warmenhuizen, The Netherlands; 3 Present Address: Aardevo B.V., Johannes Postweg 8, 8308 PB Nagele, The Netherlands; 4 Present Address: La Fédération Nationale des Producteurs de Plants de Pomme de Terre (FN3PT), Agrocampus Ouest, UMR IGEPP, 29260 Ploudaniel, France

## Abstract

Self-compatible (SC) diploid potatoes allow innovative potato breeding. Therefore, the *Sli* gene, originally described in *S. chacoense*, has received much attention. In elite *S. tuberosum* diploids, spontaneous berry set is occasionally observed. We aimed to map SC from *S. tuberosum* origin. Two full-sib mapping populations from non-inbred diploids were used. Bulks were composed based on both pollen tube growth and berry set upon selfing. After DNA sequencing of the parents and bulks, we generated *k*-mer tables. Set algebra and depth filtering were used to identify bulk-specific *k*-mers. Coupling and repulsion phase *k*-mers, transmitted from the SC parent, mapped in both populations to the distal end of chromosome *12*. Intersection between the *k*-mers from both populations, in coupling phase with SC, exposed a shared haplotype of approximately 1.5 Mb. Subsequently, we screened read archives of potatoes and wild relatives for *k*-mers specific to this haplotype. The well-known SC clones US-W4 and RH89-039-16, but surprisingly, also *S. chacoense* clone M6 were positives. Hence, the *S. tuberosum* source of SC seems identical to *Sli*. Furthermore, the candidate region drastically reduced to 333 kb. Haplotype-specific KASP markers were designed and validated on a panel of diploid clones including another renown SC dihaploid G254. Interestingly, *k*-mers specific to the SC haplotype were common in tetraploid varieties. Pedigree information suggests that the SC haplotype was introduced into tetraploid varieties via the founder “Rough Purple Chili”. We show that *Sli* is surprisingly widespread and indigenous to the cultivated gene pool of potato.

**Electronic supplementary material:**

The online version of this article (10.1007/s00122-020-03627-8) contains supplementary material, which is available to authorized users.

## Introduction

Despite potato being a major food crop, genetic gains for yield have been insignificant over the last century (Douches et al. 1996), while for maize yield increases of 1% per year have been achieved (Duvick 2005). This lack of progress in breeding is embodied by century old varieties, such as Russet Burbank and Bintje, still widely grown today (Lindhout et al. 2011). Sexual reproduction of potato is characterised by polyploidy, tetrasomic inheritance of multiple alleles and inbreeding depression upon selfing. Tetraploids can be selfed, but in conventional breeding this is uncommon. Hence, conventional potato breeding by crossing two highly heterozygous tetraploid clones is a numbers game, and the probability to find progeny with many combinations of beneficial alleles is small. Likewise, selection against mutational load in the germplasm is hardly feasible because deleterious recessive alleles are rarely exposed. Furthermore, clonal selection takes 5–9 years (Gopal 2015) due to the destructive use of tubers for phenotyping and the modest tuber reproduction rate. More importantly, with a single meiotic event per clonal selection cycle, the amount of allelic rearrangement over time is much lower than for crops with annual sexual reproduction (Jansky and Spooner 2018). To overcome the limitations of conventional breeding, alternatives breeding schemes have been proposed. Analytical breeding, proposed by Chase (1963), involves ploidy reduction and breeding at the diploid level, followed by resynthesis of tetraploids. Recently, F1-hybrid breeding at the diploid level has been proposed (Jansky et al. 2016; Lindhout et al. 2011), where inbred lines allow fixation of genetic gains and removal of unfavourable alleles. This requires self-compatible diploid germplasm tolerating inbreeding depression. However, while tetraploid potatoes are self-compatible, at the diploid level an S-RNase-based gametophytic self-incompatibility system prevents inbreeding (de Nettancourt 1997; McClure et al. 2011).

S-RNase-based self-incompatibility (SI) is found among the *Rosaceae*, *Plantaginaceae* and the *Solanaceae* (de Nettancourt 1997; McClure et al. 2011). It is controlled by a single, highly polymorphic *S*-locus, encoding for a style specific S-RNase gene and several pollen-specific *S*-locus F-box (SLF) genes (Kao and Tsukamoto 2004). Molecularly, SI is due to the cytotoxic effect of S-RNases inhibiting pollen tube growth in the style (Lee et al. 1994; Murfett et al. 1994). On the other hand, self-compatibility (SC) results from the SLF-mediated ubiquitination of style S-RNases (Sijacic et al. 2004; Kubo et al. 2015). As predicted by the collaborative non-self-recognition model (Kubo et al. 2010), each SLF allelic variant can specifically mediate ubiquitination of some of its non-self S-RNases and the combined action of the SLFs of one *S*-haplotype mediates degradation of all except its own S-RNases (Sun et al. 2018). Hence, SI occurs when the *S*-haplotype of the pollen matches either of the two *S*-haplotypes of the style. In potato, the *S*-locus is located on chromosome *1* (Gebhardt et al. 1991; Jacobs et al. 1995) within a region of low recombination, consistent with the hypothesis that SI can only be maintained in the absence of recombination between S-RNases and SLFs (Kubo et al. 2015). Tetraploids are SC because their diploid pollen is heteroallelic. The expression of two different sets of SLFs enables mutual weakening or competitive interaction (Kubo et al. 2010) resulting in the ubiquitination of all S-RNases.

SC diploid clones can be engineered by the introduction of an extra *SLF* gene to induce competitive interaction, or by knoc*k*-out of the S-RNase (Ye et al. 2018; Enciso-Rodriguez et al. 2019) or other essential genes such as *HT* (Kondo et al. 2002). Alternatively, SC can be introgressed from natural mutants since SC clones have been spotted among several SI relatives of potato (Cipar et al. 1964). For instance, Zhang et al. (2019b) recently described the *S. stenotomum* landrace Huasa Amarilla (a.k.a. C151 or CIP 705468) as SC. The most well-known source conferring SC is the dominant *Sli* (*S*-locus inhibitor) gene originally identified in chc 525-3, a clone of the typically self-incompatible species *S. chacoense* (Hosaka and Hanneman 1998a). Subsequent hybridisation between chc 525-3 and *S. phureja* was used to introduce SC in cultivated potato (Phumichai and Hosaka 2006). Seven generations of inbreeding of another *Sli*-bearing *S. chacoense* clone lead to the selection of the highly homozygous clone M6 (previously known as chc 523-3) (Jansky et al. 2014). SC is also found in *S. tuberosum* germplasm. So far, three clones have been described in the literature: US-W4 (De Jong and Rowe 1971) and G254 (Olsder and Hermsen 1976), dihaploids extracted from Minn. 20-20-34 and Gineke, respectively, and the diploid clone RH89-039-16 (Peterson et al. 2016). Interestingly, in three independent mapping studies, the distal end of chromosome *12* was associated with self-compatibility, or self-fertility: (1) the *Sli* gene of chc 525-3 (Hosaka and Hanneman 1998b); (2) fruit set upon selfing in clone RH89-039-16 (Peterson et al. 2016); and (3) a major QTL for SC using clone CD-320-20 (Gardner et al. 2019). Clone CD-320-20 is derived from US-W4 (personal communication Dr. Walter S. De Jong, Cornell). Innovation of potato breeding would benefit from broadening the gene pool of self-compatible germplasm with good agronomical traits. In our diploid breeding programme, we recognised plants with spontaneous berry set which could represent new sources of SC. Therefore, it seems prudent to characterise these additional SC sources from potentially different genetic backgrounds.

As whole genome sequencing (WGS) becomes more affordable, mapping by DNA sequencing of bulks has been applied successfully to identify the causal loci of various traits of interest (Schneeberger et al. 2009; Tribhuvan et al. 2018; Wu et al. 2018). The recently developed Comparative Subsequence Sets Analysis (CoSSA), based on sub-reads (*k*-mers) and set algebra, has proven its efficiency and robustness to identify haplotype-specific SNPs linked to a potato wart resistance gene (Prodhomme et al. 2019). The use of pedigree information allows to confirm haplotype specificity of sequence variants via clones identical-by-descent (IBD) and to trace back the origin of specific haplotypes. In this study, we used the CoSSA workflow to map SC in two segregating diploid potato populations. Subsequently, we developed haplotype-specific KASP markers for marker-assisted breeding. Finally, sequencing data were used to compare various SC clones and to trace back the origin of SC with the help of pedigree information.

## Materials and methods

### Plant material

Two diploid *S. tuberosum* mapping populations were developed: IVP16-587 and IVP17-618, both segregating for SC (pedigree information shown in ESM1). During previous growing seasons, spontaneous berry set was observed on IVP11-389-22, the female parent of population IVP16-587 and on IVP06-158-2 the male parent of population IVP17-618. Both mapping populations were sown in July 2018, and 100 vigorous seedlings per population were transplanted in five-litre pots in an open ground greenhouse compartment with drip irrigation and wet pad-and-fan evaporative cooling system.

### Phenotypic observations

Phenotypic data were collected on pollen fertility, female fertility, pollen tube growth in the style, berry set and seed set. Pollen fertility was examined by light microscopic estimating the percentage of stainable pollen using 0.2% acid fuchsine (1% fuchsine w/v glacial acetic acid 12.5%, 25% water, 67.5% glycerol). During a 6 weeks flowering period, every other day, up to three flowers per plant were selfed, until at least five up to 13 flowers were selfed. Other flowers were used to evaluate male and female fertility with testcrosses using unrelated diploids with known male and female fertility to distinguish infertility from self-incompatibility. The number of berries was recorded after 8 weeks. Seed set in ripened fruits was examined to rule out parthenocarpic development. The amount of seed per berry was visually estimated on an arbitrary scale from 1 (few) to 3 (many) by cutting three berries per plant.

**Fig. 1:**
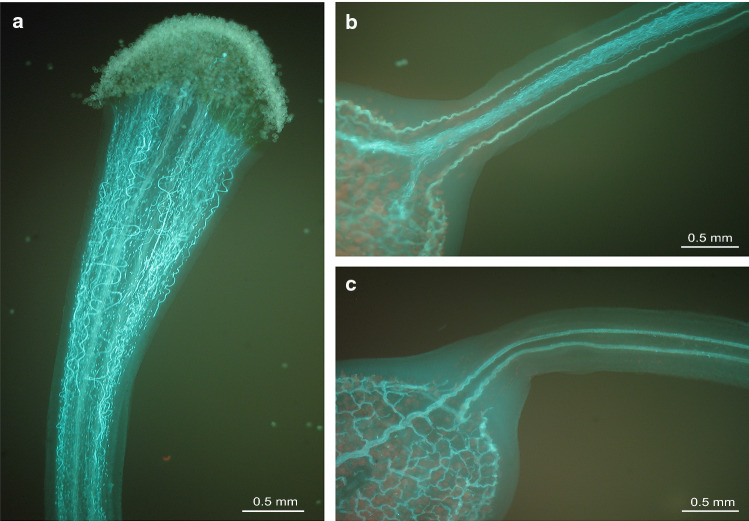
Pollen tube growth in style. **a** Pollen tube growth from stigma to style. **b** Ovary end of a self-compatible clone with pollen tubes reaching the ovary. **c** Ovary end of a self-incompatible clone without pollen tubes reaching the ovary

Pollen tube growth in the style and pollen tube arrest was observed under UV microscope with a DAPI filter (excitation filter 365 nm; dichroic mirror 425 nm; emission filter 470 nm). In three replications, styles were collected 48 h after selfing. The fixation and staining protocol followed Peterson et al. (2016). Images were taken with the Zeiss Axiocam 305 colour camera to record the amounts and growth of pollen tubes in the style near the stigma and near the ovary (Fig. 1). This was scored on an arbitrary scale from 0 to 3 as follows: no pollen tube, few pollen tubes (< 20), some pollen tubes, many pollen tubes. Plants with few pollen tubes near the stigma, or inconsistent scores between biological replicates were excluded from evaluation.

Kendall’s Tau correlation (*τ*) was calculated for the phenotypic observations described above. Plants were regarded as SC when a substantial amount of pollen tubes reached the ovaries and resulting in more than 75% berry set. SI was concluded when all (or occasionally when almost all) tubes showed growth arrest in the style and none reached the ovaries. This resulted in bulking 10 SC and 11 SI plants for population IVP16-587, and 12 SC and eight SI plants for population IVP17-618.

### Genotyping

To identify the SC locus, we followed the bulked segregant analysis method (BSA; Michelmore et al. 1991). Vigorous offspring yielding berries with seeds and without stylar pollen tube arrest were selected and pooled in the SC bulk. Offspring with stylar arrest of pollen tube growth, berry drop upon selfing and berry set upon crossing with fertile testers were pooled in the SI bulk. This results in four DNA samples per mapping population indicated as PSC and PSI representing the two parental lines and BSC and BSI representing the bulks. The samples BSC and BSI were created by pooling equal amounts of young leaf tissue. DNA was extracted with DNeasy 96 Plant Kit (QIAGEN, Venlo, NL) and from each sample, 25 Gb of clean sequences (100 bp paired-end small insert) were ordered from BGI (Copenhagen, DK) according to manufacturer’s conditions. The sequencing data generated are available from the European Nucleotide Archive (ENA) under the BioProject ID PRJEB36551.

Read archives of SC diploid clones RH89-039-16, M6 and US-W4 were retrieved from the ENA (BioProject ID PRJEB2504, PRJNA362370 and PRJNA356643, respectively). Pair-ends runs ERR033768, ERR033769, ERR033770, ERR033771, ERR033772 and ERR033773 were used for RH89-039-16; SRR5264017, SRR5264021 and SRR5264022 were used for M6; and SRR5090798 for US-W4. The read archive of Hardigan et al. (2017), describing DNA sequence diversity of wild tuber-bearing Solanum species, landraces and American varieties, was retrieved from BioProject PRJNA378971. The *Solanum phureja* DM1-3 (DM) pseudomolecule DM v4.03 was retrieved from Spud DB (http://solanaceae.plantbiology.msu.edu/).

Bulk-specific *k*-mers were identified with the CoSSA strategy (Prodhomme et al. 2019; https://github.com/cprodhom/CoSSA-workflows). In short, sequence reads were quality trimmed and adapter sequence where removed using Trimmomatic (v0.32) (LEADING:3, TRAILING:3, *SLI*DINGWINDOW:4:15 and MINLEN:70) (Bolger et al. 2014). The Glistmaker program of the GenomeTester4 toolkit (Kaplinski et al. 2015) was used to build *k*-mer tables for each sample in this study. A *k*-mer size of 31 nucleotides was selected, compromising between sequence uniqueness and sequence correctness. *K*-mers observed with a frequency of one were removed from the data set as they were likely caused by sequencing errors. The GlistCompare program of GenomeTester4 was then used to perform set operations such as union, intersection or differences.

**Fig. 2:**
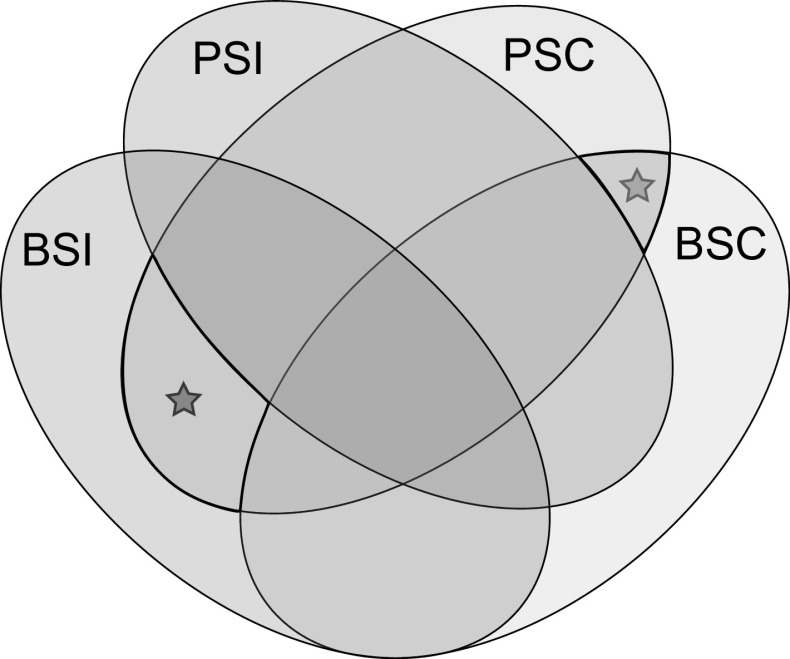
Set operations shown as Venn diagram. Intersections of *k*-mers from the SC (blue oval) and SI parent (red oval), and the SC (green oval) and SI bulk (orange oval) result in identification of *k*-mers associated with SC in coupling phase (green star) or repulsion phase (blue star) (color figure online)

K-mers in coupling and repulsion phase with SC were obtained using set operations, as illustrated in Fig. 2, and the resulting volumes of *k*-mers are reported in ESM2. Hereafter, we use the A\B and A∩B notation to indicate the set difference (elements specific to A) and the intersection of elements shared between the sets A and B.

*K*-mers in coupling phase with a dominant SC locus must be specific to the SC bulk and transmitted from the SC parent. They are obtained from the intersection between SC bulk-specific and SC parent-specific *k*-mers. Using set algebra notation, those operations can be summarised as (BSC\BSI)∩(PSC\PSI)). *K*-mers in repulsion phase with the SC locus must be specific to the SI bulk and transmitted from the SC parent. Assuming that the haplotype in repulsion phase with the SC locus in the SC parent is not shared with the SI parent, the *k*-mers linked in repulsion phase are obtained via the operation (BSI\BSC)∩(PSC\PSI).

Tables of *k*-mer spectra (reported in ESM3) were mapped to DM v4.03 using BWA aln (v0.7.12) allowing 3 mismatches. The number of *k*-mers per 1 Mb bins, counted using bedtools (v2.25) (Quinlan and Hall 2010), were plotted using Matplotlib (Hunter 2007).

*K*-mer analysis was performed independently for mapping populations IVP16-587 and IVP17-618. The sets of *k*-mers in coupling phase with the SC locus of IVP16-587 and IVP17-618 (IVP16/17SCcoup) were compared to assess if the two mapping populations shared the same SC haplotype. The resulting table was depth refined, mapped and plotted as described above.

### Comparison with reads archives

To investigate the origin of the SC haplotype, we used this IVP16/17SCcoup *k*-mer table to assess the presence of the SC haplotype-specific *k*-mers in well-known SC diploid clones M6, RH89-039-16, US-W4 and C151, and in a panel of potato varieties, landraces and wild relatives (Hardigan et al. 2017). To do so, we computed the intersection between our IVP16/17SCcoup *k*-mers and the *k*-mers generated from these read archives (ESM4). The resulting *k*-mers tables were depth refined and mapped as described above. The number of *k*-mers per bins of 50 kb was counted and plotted using seaborn (Waskom et al. 2014). Alternatively, mapped *k*-mers were directly visualised using Integrative Genome Viewer v.2.5.3 (IGV) (Robinson et al. 2011). Finally, the online potato pedigree database (van Berloo et al. 2007) was used for the familial interpretation of our data.

### Estimation of genetic distances

Genetic distances between clones were estimated using the Mash algorithm version 2.2 (Ondov et al. 2016). Mash distances resemble Jaccard index between *k*-mers sets and correlate well with the average nucleotide identity. Trimmed reads were used as input with default sketches parameters and 31-mers. The distance matrix obtained was used in a principal coordinate analysis (PCoA) and plotted with Past version 3.26 (Hammer et al. 2001).

## Results

### Analysis of SC-related phenotypic data and bulk composition

Out of the 100 plants grown in populations IVP16-587 and IVP17-618, 57 and 47 plants were excluded from analysis, respectively, because of poor vigour or because less than nine flowers could be selfed. Phenotypic data on berry set, amounts of seed set, pollen viability and amounts of pollen tube growth were collected on the remaining plants (ESM 5).

Kendall’s Tau correlations between pollen stainability, amount of pollen tubes in the style near the stigma, berry set and seed set were weak and τ never exceeded 31%. In contrast, the amount of pollen tubes in the style near the ovary correlated strongly with berry set (*τ* = 0.64 and 0.69 in population IVP16-587 and IVP17-618, respectively). Berry set correlated reasonably well with the amounts of seed per berry (*τ* = 0.60 and 0.51). This is consistent with the observation that parthenocarpic fruit development was not frequent and typically observed only when flower drop (no berry set) was already high.

Among the 43 remaining plants of IVP16-587, 15 plants did not set any berry and were considered SI, while 13 plants with more than 75% of berry set were considered SC. Hence, fifteen plants were inconclusive based on berry set alone. Among the 53 remaining plants in IVP17-618, 14 plants did not set any berry and were considered SI, while 11 plants with more than 75% of berry set were considered SC, leaving 28 plants inconclusive based on berry set alone. For both populations, we find no reason to reject a 1:1 Mendelian segregation for SC or SI, when inconclusive plants are ignored. We used the assumption that SC inherits as a monogenic dominant trait, in our subsequent strategy to bulk samples and to perform and analyse k-mer operations.

In addition to this first classification based on berry set, we used pollen tube arrest as an additional criterion, as well as a threshold of at least nine self-pollinated flowers. Pollen tube arrest—recorded as a strong decrease in the amount of pollen tubes from stigma to ovaries—resulted in berry drop. Likewise, unaffected amounts of pollen tubes near the ovaries resulted in berry set. Finally, our interpretation of reproduction traits resulted in the following bulks: BSC *N* = 10 and BSI *N* = 11 plants for IVP16-587; and BSC *N* = 12 and BSI *N* = 8 plants for IVP17-618.

### Comparative Subsequence Sets Analysis (CoSSA) to identify haplotype-specific SNPs associated with self-compatibility

DNA sequencing of the SC and SI parents and SC and SI bulks (PSC, PSI, BSC, BSI) of both populations yielded between 27 and 29 Gb of sequence data for each sample (reported in EMS2). Considering a haploid genome of 844 Mb, this represents a read depth of ~ 34 × per diploid parent or 17× per haploid genome. Assuming a uniform sequence coverage, the expected depth of 31-mers per haploid genome is ~ 12× and agreed well with observed *k*-mer spectra (reported in ESM3). Hence, tables of *k*-mers associated in coupling and repulsion phase with SC can be refined by selecting for a depth between 6× and 23×. We followed CoSSA (Prodhomme et al. 2019) to identify haplotype-specific SNPs via set algebra.

**Fig. 3:**
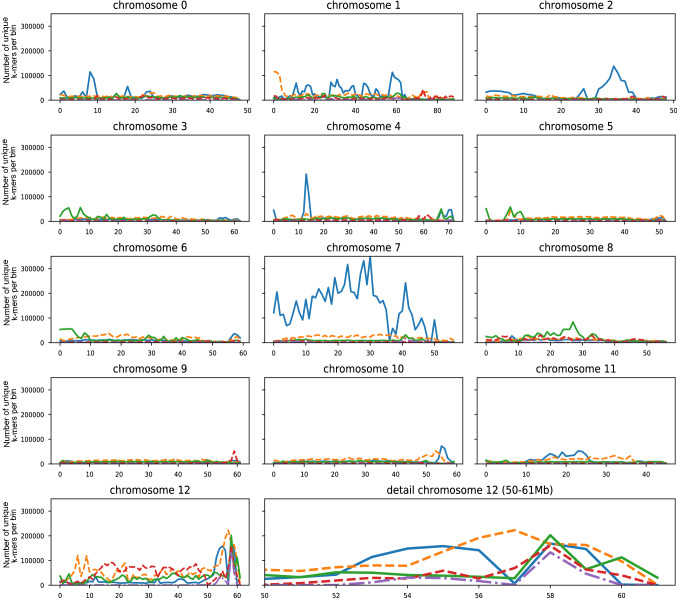
Self-compatibility mapped to the distal end of chromosome *12* using density graphs of unique *k*-mers mapped in 1 Mb bins of the reference genome DM v4.03. *K*-mers associated in coupling phase (solid blue line) or in repulsion phase with SC in population IVP16-587 (dashed orange line). *K*-mers in coupling phase (solid green line) or repulsion phase (dashed red line) with SC in IVP17-618. Intersection of coupling phase linked *k*-mers shared by IVP16-587 and IVP17-618 (dashed-dotted purple line) (color figure online)

In population IVP16-587, out of the 62,128,547 SC bulk-specific *k*-mers (BSC\BSI), 58% were transmitted from the SC parent only ([BSC\BSI]∩[PSC\PSI]). After depth filtering, we kept 20,972,831 unique *k*-mers assumed to be linked in coupling phase with a dominant SC locus. Similarly, 81,326,597 *k*-mers were unique to the SI bulk (BSI\BSC) out of which 40% were inherited from PSC only ([BSI\BSC]∩[PSC\PSI]), giving after depth filtering 17,935,863 unique *k*-mers assumed to be associated in repulsion phase with SC. From the sets of *k*-mers associated in repulsion and coupling phase with SC, 92% and 84% could be mapped to DM v4.03, respectively. In Fig. 3, the volumes of unique *k*-mers are plotted to the potato chromosomes in 1 Mb intervals.

Strikingly, 39.8% of the *k*-mers associated in coupling phase with SC mapped on a series of overlapping peaks spanning most of chromosome 7, from the proximal end to 48 Mb. Besides of chromosome *7*, the highest peak was identified on the distal end of chromosome *12*, from 53 Mb to 60 Mb, where 4.6% of the *k*-mers associated in coupling phase with SC mapped. Several smaller peaks of *k*-mers could be observed in other genomic regions, notably on chromosomes 0, 1, 2 and 4. An elevated frequency of *k*-mers linked in repulsion phase with SC was observed on the entire chromosome *12*. The highest peak was located between 55 and 61 Mbp where 6.5% of the *k*-mers mapped. Additionally, a smaller peak was observed on the first 4 Mb of chromosome *1*.

In population IVP17-618, out of the 87,512,765 SC bulk-specific *k*-mers, 31% were inherited from PSC only. After depth filtering, 11,289,401 unique *k*-mers remained, which are assumed to be linked in coupling phase with SC. Similarly, 53,170,315 *k*-mers were unique to the SI bulk out of which 30% were inherited from PSC only. After depth filtering, we kept 7,326,761 unique *k*-mers, assumed to be linked in repulsion phase with SC. From the sets of *k*-mers associated either in coupling or repulsion phase with SC, 86% and 92% could be mapped to DM v4.03, respectively, also shown in Fig. 3.

Again chromosome *12* shows high frequencies of *k*-mers in coupling phase with a peak between 58 and 61 Mbp where 3.9% of the *k*-mers mapped. Several smaller peaks were observed on chromosomes *3*, *4*, *5*, *6* and *8*. Similarly, the high frequencies of *k*-mers associated in repulsion phase with SC were observed across chromosome *12*. The highest peak was identified on chromosome *12* between 58 and 61 Mbp where 4.3% of the *k*-mers mapped. Additionally, smaller peaks could be observed on chromosomes *1*, *8* and *9*.

Overall, the CoSSA suggests various chromosomal regions potentially associated with SC. Interestingly, when comparing the distribution of *k*-mer peaks assumed to be linked in coupling phase with SC with those in repulsion phase, only the peaks on chromosome *12* overlapped. Moreover, the distal end of chromosome *12* was supported by coupling and repulsion phase *k*-mer peaks in both populations. This suggests that both populations may share the same haplotype involved in SC. To verify this hypothesis, an intersection was made between the *k*-mers in coupling phase with SC of population IVP16-587 and IVP17-618. After depth filtering, a set of 905,192 unique *k*-mers assumed to be in coupling phase the SC locus in both populations was obtained. Eighty-four per cent of those *k*-mers could be mapped to potato reference genome. The only peak observed was located on the distal end of chromosome *12* were 29.8% of the *k*-mers mapped (Fig. 3). Visualisation of the same set of *k*-mers in IVG allowed a more precise identification of the haplotype boundary shared by the two SC bulks, which ran from 58.03 Mb to 59.36 Mb (ESM 6a).

### Read archives expose the widespread presence of the haplotype conferring self-compatibility

**Fig. 4:**
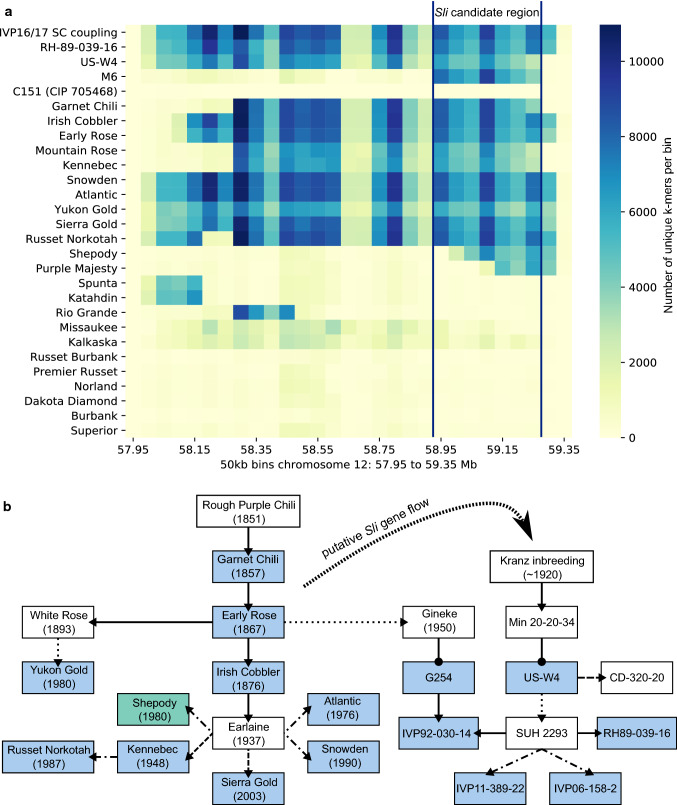
**a** Detection of high densities of specific *k*-mers associated with SC (mapped to 50 kb bins of DM v4.03; columns) across various potato genotypes (rows) suggests the identification of one common haplotype involved in SC at a distal locus on chromosome *12*. Row 4 represents the *Sli* locus of M6. This suggests that one haplotype identical-by-state to *Sli* is also present in potato varieties. Genomic sequences from well-known SC diploids and varieties (Hardigan et al. 2017) were retrieved from public archives. **b** Pedigree relationships between genotypes positive for the SC haplotype. Year of market release is shown for varieties. The first (putative) SC ancestor of each clone was annotated together with the generation at which it was found. Blue boxes: clone bearing a complete SC candidate region; teal box: clone bearing an incomplete SC candidate region; white boxes: clones not tested in this study. Relatives are connected with arrows. First generation relatives—solid arrows; 2nd generation relatives—dashed arrows; 3rd generation relatives—dashed-dotted arrows; 6th generation relative—dotted arrow. Line with dot-node: dihaploidisation (color figure online)

The identification of a locus involved in SC at the distal end of chromosome *12* is reminiscent to the position of the *Sli* locus as described by Hosaka and Hanneman (1998b), as well as the locus involved in self-fertility proposed by Peterson et al. (2016). Therefore, we compared our haplotype-specific *k*-mers with *k*-mers generated from publicly available read archives of the diploid clones M6 (representing the *Sli* locus) and RH89-039-16 used by Peterson et al. (2016). This comparative study also included other well-known diploid SC clones: US-W4 and C151, as well as a panel of varieties, landraces and wild tuber-bearing *Solanum* species sequenced by Hardigan et al. (2017). The *k*-mer volumes of the intersection between k-mers in coupling phase with SC shared by population IVP16-587 and IVP17-618 (IVP16/17 SC coupling), and these public read archives are visualised in Fig. 4a.

Each row in Fig. 4a shows a plot of the density of SC-associated *k*-mers in windows of 50 kb, as identified in each specific potato sample. A clear differentiation can be made between genotypes where the intersection identified high or low densities of shared *k*-mers. We observed that the SC clones RH89-039-16, M6 and US-W4, display high shared *k*-mer densities. This observation supports identity-by-state (IBS) of the *Sli* locus of M6 with the SC haplotype of RH89-039-16 and US-W4, as well as with the one of the SC parents of our mapping populations: IVP11-389-22 and IVP06-158-2. Interestingly, the SC clone C151 does not share *k*-mers with this SC haplotype which is in agreement with the different mechanism conferring SC in C151 (Zhang et al. 2019b).

Figure 4a shows the physical length of the SC (sub-)haplotypes as well as the positions of recombination breakpoints. While our SC-associated *k*-mers identified a long SC haplotype in RH89-039-16, *k*-mers from bin 59.35–59.40 Mb were absent in US-W4 and M6 haplotype. Interestingly, most of them were found in PI498359, a *S. kurtzianum* accession (ESM7). More importantly, the *k*-mers mapping from 58 Mb to 58.95 Mb was virtually absent in M6, suggesting a recombination breakpoint and allowing us to limit the *Sli* candidate region to seven bins of 50 kb, located from 58.95 to 59.30 Mb. Direct visualisation of *k*-mer mapped using IGV allowed us to identify recombination breakpoints at 58.945 Mb and 59.278 Mb pinpointing *Sli* to a candidate region of 333 kb (ESM 6b).

It was unexpected to recognise high densities of SC-associated *k*-mers in several tetraploid varieties, while no significant amounts of SC-associated *k*-mers were found in the landraces and wild species (ESM7) sequenced by Hardigan et al. (2017). A long SC haplotype was found in Garnet Chili, Irish Cobbler, Early Rose, Kennebec, Russet Norkotah, Sierra Gold, Yukon Gold, Snowden, Atlantic and Mountain Rose. The SC-associated *k*-mers found in Katahdin and Spunta as well as Rio Grande only mapped to bins outside of the *Sli* candidate region (58 Mb to 58.2 Mb and 58.3 Mb to 58.55 Mb, respectively). Interestingly, SC-associated *k*-mers of Shepody and Purple Majesty only mapped from 59 Mb to 59.4 Mb and 59.1 Mb to 59.4 Mb, respectively, suggesting a shorter haplotype due to recombination events within the SC haplotype in those two clones.

When sequencing depth allows it, an estimation of *Sli* dosage can be deduced by comparing the *k*-mer spectra of *k*-mers mapping to the *Sli* candidate region with the total *k*-mer spectrum of a sample. The peak of haplotype-specific *Sli k*-mers can either coincide with the simplex peak of all *k*-mers or be shifted towards higher frequency. This shift represents a higher allele dosage of *Sli*. The data presented in ESM8 suggest that possibly Garnet Chili, Irish Cobbler, Early Rose and certainly Russet Norkotah are duplex for the *Sli* allele. Elevated allele dosages are not unexpected given the prevalence of the *Sli* locus and its distal position facilitating double reduction.

The widespread observation of a region involved in SC identical-by-state in old North American varieties and contemporary diploid progenitor clones prompted the question of how these potato genotypes relate to each other. A summary of the relatedness of varieties with the SC haplotype is shown in Fig. 4b based on potato pedigree information (van Berloo et al. 2007). The SC haplotype seems to have spread among tetraploid varieties via the frequent use of Early Rose, Irish Cobbler and potentially Earlaine as parents. Because all positive genotypes are closely related, we conclude that the SC haplotype is identical-by-descent. The variety Rough Purple Chili, introduced into the USA in 1851 by Goodrich (1863), is the origin of the SC haplotype. Figure 4b also shows that all SC diploids have US-W4 as common ancestor. Subsequently, the SC haplotype spread in the breeding stock of Wageningen University via the use of the (now lost) clone SUH 2293. US-W4 is a dihaploid extracted from Minn. 20-20-34 a tetraploid progenitor with an unknown pedigree from the breeding programme of Frank Krantz in the 1920s. Therefore, it is reasonable to assume that Minn. 20-20-34 is also derived from Rough Purple Chili.

**Fig. 5:**
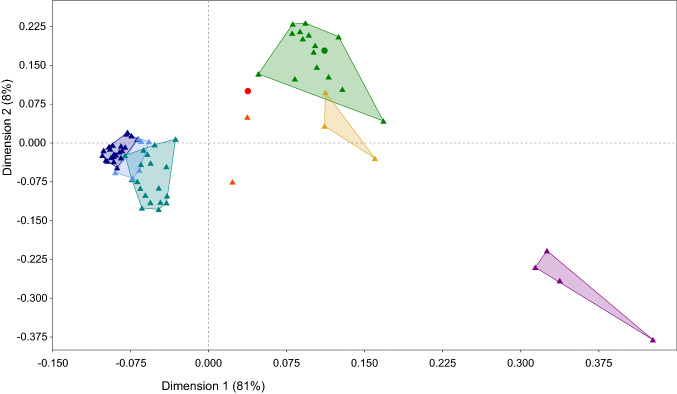
Principal coordinate analysis (PCoA) between the *Solanum* clones based on Mash distance. Symbol colours are based on clusters identified before by Hardigan et al. (2017; Fig. 1, data set 1). Wild species outgroup (purple triangles), wild species (green triangles), wild subgroup diverging from the cultivated lineage after most other species (gold triangles), landraces (teal triangles), tetraploid varieties (navy triangles); with the addition of hybrid between wild species and landraces (orange triangles) and diploid *S. tuberosum* (cornflower blue triangles). M6 is represented by a red dot and *S. chacoense* (PI 275139) by a green dot (color figure online)

Remarkably, high densities of SC-associated *k*-mers, signature of the SC haplotype identified in this study, were also identified in clone *S. chacoense* M6. A shared haplotype between a wild and cultivated potato clones can be explained by introgression or coancestry. To test these hypotheses, the genetic distance was estimated between the clones used in this study and those sequenced by Hardigan et al. (2017) using Mash (Ondov et al. 2016). As expected, *S. chacoense* accession PI-275139 is most closely related clone to M6. However, while *S. chacoense* PI-275139 is genetically close to *S. berthaultii, S. infundibuliforme, S. boliviense* and various *S. brevicaule* accessions; M6 shares a low Mash distance with several *S. tuberosum* tetraploid varieties (Table 1). Moreover, when the pairwise distance matrix of all clones was used in a PCoA (Fig. 5), M6 clustered between varieties and landraces and a cluster of wild species. In addition, M6 seems to cluster with *S. candolleanum* sp. *multidissectum* (PI 210044) and *S. tuberosum* sp. *stenotomum* (PI 195204), both described as hybrids between cultivated landraces and wild species (Hardigan et al. 2017). These results suggest that M6 is not a pure *S. chacoense*.

**Table 1:** Top 10 genetically nearest clones of *S. chacoense* (PI 275139) and M6 based on genetic distance estimates using Mash software

*S. chacoense* (PI 275139)	Mash distance	M6	Mash distance
M6	0.0235827	*S. chacoense* 2×	0.0235827
*S. berthaultii* 2× (PI 458365)	0.0254441	Dakota Diamond tuberosum 4×	0.0249416
*S. infundibuliforme* 2× (PI 458324)	0.0281594	Snowden tuberosum 4×	0.0257861
*S. boliviense* 2× (PI 545964)	0.0283536	Premier Russet tuberosum 4×	0.0259593
*S. brevicaule*–*sparsipilum* 2× (PI 473385)	0.0286483	Rio Grande Russet tuberosum 4×	0.0261340
*S. brevicaule*–*gourlayi* 2× (PI 473065)	0.0286483	Sierra Gold tuberosum 4×	0.0269388
*S. brevicaule*–*brevicaule* 2× (PI 498112)	0.0289472	Mountain Rose tuberosum 4×	0.0269388
*S. brevicaule*–*spegazzinii* 2× (PI 472978)	0.0299747	Burbank tuberosum 4×	0.0270302
*S. verrucosum* 2× (PI 275260)	0.0302927	Yukon Gold tuberosum 4×	0.0271220
*S. brevicaule*–*leptophyes* 2× (PI 545987)	0.0302927	Irish Cobbler tuberosum 4×	0.0271220

### Haplotype specificity of k-mers

Only for IVP11-389-22 and IVP06-1582, the SC mapping parents of this study, it is assumed that all SC-associated *k*-mers are haplotype specific and thus linked in coupling phase. However, in the panel sequenced by Hardigan et al. (2017), *k*-mers can originate from homologous chromosomes and represent a blend of haplotypes. If pedigree information is true and Early Rose is a direct descendant of Garnet Chili, then *k*-mers mapping from bins 58.10 to 58.25 Mb and in bin 59.30 Mb, absent in Garnet Chili, must originate from the pollinator chromosome(s). Subsequent recombination must have brought those *k*-mers in coupling phase in IVP11-389-22 and IVP06-158-2, the parents of our mapping populations. Similarly, *k*-mers from Russet Norkotah which could be mapped from 58 to 58.15 Mb and 58.3 to 59.3 Mb might be on different homologues. Two recombination events in bins 58.20 and 58.25 Mb are a less probable explanation of this *k*-mer pattern.

A striking feature shown in Fig. 4 is the similarity of the pattern of the pseudo-colours which reflect variations in *k*-mer densities among rows positive for the SC haplotype. These variations reflect the genomic landscape of the reference genome where unique or low-complexity DNA enabled mapping of many or few *k*-mers, respectively. These patterns contain no information on IBD. For instance, the low frequency of *k*-mers in mapping between 58.65 and 58.75 Mb as well as between 58.85 and 58.95 Mb can be explained the 5000 bp gaps between DM scaffolds PSGC0003DMB000000034 and PSGC0003DMB000000750 as well as between and PSGC0003DMB000000750 and PSGC0003DMB000000114. Overall, IBD is concluded on the basis of the congruence between the rows, but not on the shade of the colour itself.

### Validation of CoSSA results and development of MAS tools

To verify our in silico result biologically, haplotype-specific KASP markers were designed both to verify the SC haplotype on chromosome *12* as well as to disprove large *k*-mers identified in coupling phase with SC during CoSSA of IVP16-587 and IVP17-618. We tested those markers on a set of 93 diploid clones hereafter referred to as the validation panel. The validation panel was composed of the bulked and parental clones of our two mapping populations, a subset of two parental lines and five progenies from a full-sib population IVP16-560 and 37 distantly related diploids with well-known SC or SI phenotype or tested for seed set upon self-pollination. The results are illustrated in ESM9.

No association was observed with SC for the 27 KASP markers at the 10 population specific *k*-mers peaks beyond chromosome *12*, confirming the tentative conclusion that those other peaks observed were false positive. All 18 KASP markers designed within the SC candidate region on chromosome *12* allowed a perfect prediction of SC in populations IVP16-587 and IVP17-618, albeit few missing values. Hence, CoSSA results could be confirmed with KASP markers.

Another full-sib population IVP16-560, studied for self-fertility in prior unpublished work, showed a seven self-infertile versus 83 self-fertile segregation, severely deviating from the expected 1:1 ratio. The five IVP16-560 offspring clones tested with KASP (three infertile and two fertile clones) are heterozygous for all 18 KASP markers, and the SC parent IVP92-030-14 is homozygous. Marker data explain the lack of segregation for SI/SC in IVP16-560, because the SC parent IVP92-030-14 is homozygous for the SC haplotype and thus generated a uniformly heterozygous SC progeny. The seven self-infertile descendants observed in IVP16-560 did not set berries, not because of SI, but rather because of poor fertility.

KASP genotyping of the rest of the validation panel, composed of unrelated clones from our diploid collection, showed a correlation between the KASP markers and SC phenotype for 30 out of 40 clones. Nine clones positive for SC specific KASP alleles did not set berries due to lack of viable seeds after selfing or due to lack of fertility. One clone yielded 25 seeds after selfing despite the absence of SC specific KASP alleles. Contamination during pollination or DNA sampling/extraction errors may explain this contradiction.

Interestingly, the validation panel included RH89-039-16, G254 and two SC clones raised from *S. chacoense* seedlings: 07-2N100-1-5 and 07-2N104-2 (kindly provided by dr. Kazuo Hosaka). KASP results for these four clones indicated the presence of the SC haplotype and confirm IBS for *Sli* from *S. chacoense* and the SC haplotype observed in *S. tuberosum* clones. The SC of the dihaploid clone G254 (Hermsen 1978), extracted from the tetraploid variety Gineke, can now be explained, because Gineke is a descendant of Early Rose (Fig. 5), so IBD is confirmed between the SC haplotype of G254 and Rough Purple Chili.

## Discussion

### Pollen tube growth is most indicative for SC

Spontaneous berry set is rarely observed in diploid potato, but the lack of successful sexual reproduction upon selfing has many reasons apart from self-incompatibility, such as infertility of male or female gametes, lack of gamete fusion and embryo abortion (reviewed by Johnson et al. 2019) Vegetative crops are subjected to low selection pressure for fertility traits, and only a few tetraploid varieties are known for a high level of spontaneous berry set such as Cara, Désirée and Gineke (see: https://europotato.org/characters/view/Berries). In diploids, deleterious recessive mutations are more easily exposed, and thus, ploidy reduction will aggravate fertility problems. Nevertheless, it was commonly assumed that SC diploids are rare and SI the major reason for the inability to self-fertilise diploids. Initially, our phenotypic observations were also focussed on berry set, because this simple visual key allows classifying descendants in a segregating population easily, provided that one parent is renowned for spontaneous berries. However, berry set upon selfing is an observation related to self-fertility rather than self-compatibility (Peterson et al. 2016). To avoid mapping loci involved in fertility or parthenocarpy, we performed comprehensive phenotyping of many reproductions related traits. We found low to moderate correlations between these traits and the ability to set berries. The highest correlation was observed between berry set and amount of pollen tube reaching the ovaries. Gametophytic self-incompatibility is caused by S-RNase activity in stylar tissue, and therefore, stylar observations should allow to distinguish SI/SC better than indirect or confounding traits. However, in our experimental conditions, even the pollen tube arrest phenotype shows quantitative variation. Based on current experience, we recommend recording both pollen tube growth and berry set to unambiguously classify SC and SI plants.

Peterson et al. (2016) also started research on SC because several descendants of DM × RH89-039-16 set berries upon selfing. Surprisingly, segregation for pollen tube arrest could not be observed in their F1 population. Peterson et al. (2016) focused on phenotyping self-fertility upon inbreeding and concluded that the south of chromosome *12* was responsible for fruit set in self-fertile plants in the F1 generation, whereas other genomic regions contributed to the ability of S3 plants to set fruit after self-pollination. In this study, we proved that diploid clone RH89-039-16, bears *Sli* at a position which is in agreement with their map position at the distal end of chromosome *12*. Hence, we argue that Peterson et al. (2016) mapped self-compatibility in the F1 and identified a valuable QTL associated with self-fertility and/or inbreeding tolerance in the S3.

### The benefits of *k*-mer-based analyses

Several reasons support the Comparative Subsequence Sets Analysis of DNA sequences (Prodhomme et al. 2019). In the first place because this approach does not rely on a reference genome for variant calling. Previous mapping studies have speculated on the molecular cause of SC. Olsder and Hermsen (1976) originally favoured a translocation of the *S*-locus, which resulted in competitive interaction. Subsequent results Thompson et al. (1991) suggested a translocation of the pollen part of the S-locus only, now known as SLFs genes (Kao and Tsukamoto 2004). Alternatively, an independent inhibitor allele active in the pollen only was also proposed (Thompson et al. 1991; Hosaka and Hanneman 1998a). Translocation events may result in unreliable read mapping and also complicate the interpretation of mapping studies due to pseudo-linkage (Farré et al. 2011). As a result, reference free sequence comparison method was favoured to map the locus involved in SC.

A second reason to employ CoSSA is computational simplicity. Demanding bioinformatics steps such as read mapping and variant calling could be omitted. Instead, set algebra allows fast and stringent comparisons between samples to retain small subsets of *k*-mers.

Thirdly, the k-mers identified by CoSSA (Prodhomme et al. 2019) represent haplotype-specific SNPs, associated in coupling phase or repulsion phase with SC. Initially, the data from mapping population IVP16-587 identified a large number of *k*-mers that mapped to chromosome *7*. This peak could be ruled out because *k*-mers associated in repulsion phase with SC only confirmed the peak on chromosome *12*. This a great advantage of diploid organisms, where the accuracy of CoSSA greatly benefits from the use of repulsion phased *k*-mers. This aspect was not mentioned in the original paper, where tetraploids were used (Prodhomme et al. 2019). Haplotype specificity of markers (*k*-mers), initially obtained by BSA, can be improved by subtracting *k*-mers from any other potato clone without the trait-specific haplotype.

### Marker-assisted breeding

The haplotype-specific *k*-mers obtained with CoSSA were easily converted into KASP marker assays following recommendations by Prodhomme et al. (2019). This allowed validation of the candidate region on the distal end of chromosome *12* and to disprove the false positive *k*-mer peaks, in particular the peak on chromosome *7*. The KASP markers are available for breeders and researchers to apply marker-assisted selection of SC. This will improve the efficiency of breeding programmes, because time-consuming phenotyping of SC and distinguishing it from self-fertility can now be circumvented. Markers will be particularly beneficial for the selection of progenitors with the SC haplotype, including tetraploid progenitors used for the extraction of SC diploids, during the early stage of diploid breeding programmes. Once breeding with SC diploids makes progress, the value of marker-assisted selection will reduce. Indeed, selfing SC clones will result in 100% SC offspring, because only the pollen tubes bearing the *Sli* gene will be able to reach the ovary and produce offspring. This will result in a rapid fixation of *Sli* in homozygous state.

### Is SC associated with lethality?

Interestingly, both Hermsen (1978) and Hosaka and Hanneman (1998a) reported a recessive lethal allele linked to *Sli* inducing skewed segregation for the SC. Similarly, distorted segregation of markers was observed on the distal end of chromosome *12* in RH89-039-16 derived populations (Peterson et al. 2016; van Os et al. 2006; Zhang et al. 2019b), and in the F_2_ of DM × M6 (Endelman and Jansky 2016). Recently, Endelman et al. (2019) suggested that the lethal allele linked to *Sli* was also present in DM in the interval 57.2–57.8 Mb of chromosome *12*. The position of this region, about 1 Mb south of the *Sli* candidate region identified in this study, is consistent with the hypothesis stating that *Sli* is linked to a lethal allele. In our KASP validation panel, we identified seven putative *Sli* homozygous clones. The observation of homozygosity is not only based on KASP markers. One of the homozygous clones, IVP92-030-14, who received one *Sli* allele from G254 and the other from US-W4 (Fig. 5), only produced SC offspring. This confirms that KASP marker data are reliable predictors of the genotype at the *Sli* locus and the resulting phenotype. Hemizygosity at the *Sli* locus can be excluded as well. Therefore, it seems that the lethal allele described above has recombined from *Sli* and is not a limitation to reach homozygosity in this region. This is in agreement with Marand et al. (2019) who did not identify recalcitrant heterozygosity in the distal end of M6 chromosome *12*.

### Allele mining from read archives

Increasingly, the FAIR (Wilkinson et al. 2016) availability of nucleotide read archives allows allele mining (Van De Weg et al. 2004). Allele mining implies the identification of alleles present in the gene pool where IBD is utilised to express the identity of specific alleles in terms of alleles of founding cultivars. With set algebra, this study takes allele mining to a next level, because we directly evaluated the intersection between our haplotype-specific *k*-mers with *k*-mers from potato varieties sequenced before. Furthermore, this study demonstrates the scalability of this strategy because it circumvents read mapping and variant calling. The large data set generated by (Hardigan et al. 2017) appeared to be very useful for mining of the *Sli* allele. From the nucleotide archive of clone M6, we could identify a recombinant haplotype and narrow the candidate region of the *Sli* locus from 1.5 Mb to 333 kb. Interestingly, recombination breakpoints within the SC candidate region were also identified in the haplotype of Shepody and Purple Majesty. Fertile dihaploids derived from those varieties could be used for recombinant analysis to fine map the *Sli* locus. With the increasing amounts of sequencing data submitted to online repositories, these *k*-mer-based set operations will gain in relevance and power.

### Rough Purple Chili as founder clone of SC germplasm

Rough Purple Chili, identified as the source of *Sli* in this study, was described by Hawkes (1979) as one of the four introductions event establishing the genetic foundation of all European and North American varieties. Rough Purple Chili can be found in the pedigree of nearly all American (Plaisted and Hoopes 1989) and European varieties (Hawkes 1979). Descendants of Rough Purple Chili, such as Garnet Chili, Early Rose and Irish Cobbler are recognised as major contributing ancestors (Love 1999). As they were positive for *Sli*, it is clear that these varieties facilitated the spread of *Sli* into the American gene pool. Bradshaw et al. (2006) mentioned that Early Rose was also widely used by European breeders. For instance, Early Rose was the female parent of Abundance and Epicure, from the Scottish Plant Breeding Station. Interestingly, the dihaploid B16 induced from the clone Black 4495, bred in the same station, was described as self-compatible by Olsder and Hermsen (1976). In the meantime, Early Rose was also used as female parent in Germany to produce Imperator, an ancestor of Gineke, from which the SC dihaploid G254 (Olsder and Hermsen 1976), found positive for *Sli* KASP markers in this study, is derived. Hence, the *Sli* locus may be surprisingly common in both American and European germplasm.

### Thoughts on the origin of *Sli*

The identification of a SC haplotype, indistinguishable between historical *S. tuberosum* cultivars and *S. chacoense* clones, is not easily understood. Interspecific gene flow between cultivated and wild potato has been extensively reported (Rabinowitz et al. 1990; Celis et al. 2004; Scurrah et al. 2008; Hardigan et al. 2017) and interploidy genetic exchange is facilitated by the production of unreduced gamete in diploid potato (Watanabe and Peloquin 1989). *Sli* may be another example of geneflow between wild and cultivated potato germplasm. Based on our data, the direction and timeframe of a putative introgression of *Sli* remain speculative. Hybridisation may have occurred between the Chilean *S. tuberosum* ancestor of Rough Purple Chili and a wild *S. chacoense*. Alternatively, a more recent hybridisation may have happened between SC *S. tuberosum* dihaploids and the ancestor of M6.

A final example of the utility of k-mers was offered by the distance estimation software Mash (Ondov et al. 2016). Because all k-mer tables were already generated this software allowed to explore the question on the origin of the *Sli* locus in *S. chaocense* clone M6. Despite being an estimation, the genetic distances calculated by Mash with *k*-mers agreed very well with the classification by Hardigan et al. (2017) who used read mapping and variant calling. The wild outgroup, the wild potato species, the landraces and tetraploid varieties are properly separated by Mash distance. Hardigan et al. (2017) also identified a wild subgroup diverging from the cultivated lineage, composed of three Peruvian species (*S. medians, S. megistacrolobum and S. raphanifolium*). Based on Mash distance, the separation of this subgroup from the wild species cluster is not clear. Interestingly, the hybrids *S. candolleanum* sp. *multidissectum* (PI 210044) and *S. tuberosum* sp. *stenotomum* (PI 195204) are clustering with M6 between landraces and wild species in this study (ESM10). This was also reported by Hardigan et al. (2017) in Supplementary Fig. 6.

Remarkably, M6 shares a close Mash distance with Dakota Diamond and Snowden. Those cultivars have 1/8 and 1/16 *S. chacoense* ancestry, respectively. However, in Fig. 5, Dakota Diamond and Snowden clearly cluster with the rest of the tetraploid varieties. On the other end, M6 is localised between wild species and tetraploid varieties, suggesting that despite its putative hybrid status, M6 present a much larger proportion of *S. chacoense* ancestry than those clones. Recently, genomic data on structural variation were used to compare several landraces and M6 (Kyriakidou et al. 2019). The position of M6, clustering with the *S. tuberosum* landrace CIP 705053 once more, suggests that an ancestor of M6 hybridised with *S. tuberosum*. Although the *Sli* haplotype was not observed in wild species and landraces in the panel of Hardigan et al. (2017), and hybridisation with *S. tuberosum* may explain SC in M6 more easily, we cannot exclude a second origin of *Sli* from *S. chacoense*.

### Candidate genes

Complex biological mechanisms such as gametophytic self-compatibility are usually controlled by many different genes, coding for catalytic proteins of a metabolic route or transcription factors for a signalling cascade. Because mutations in each component could be anticipated, it is difficult to speculate on the identity of the *S*-locus inhibitor gene(s) encode by the *Sli* locus. For example, in *S. lycopersicum*, breakdown of self-incompatibility is associated with a loss of function mutation in both *S*-*RNase* and *HT* genes (Kondo et al. 2002). No obvious candidate inhibitor genes or *SLF*s were found in our candidate region. However, the annotation of the DM reference genome reveals the presence of a self-incompatibility S1 family protein (PGSC0003DMG400016877 at 58.968 Mb) and a cluster of four F-box proteins (PGSC0003DMG400016863, PGSC0003DMG400016862, PGSC0003DMG400016861 and PGSC0003DMG400046496 at 59.024-59.057 Mb) that could be proposed as candidate genes.

### Implications for diploid potato breeding

This study shows that *Sli* is widespread and that many clones can be used to breed SC diploid potato, and removes the genetic bottleneck that could be experienced by breeders in the early days of diploid inbreeding. We also show that many sources of SC could be unified into a single locus known as *Sli*. Clone C151 described as SC due to a low expression level of an *S*-allele (Zhang et al. 2019b) is an alternative to *Sli*-based SC. Additionally, two artificial SC clones were recently generated by mutation of the S-RNase gene with CRISPR-Cas9 (Ye et al. 2018; Enciso-Rodriguez et al. 2019). Both authors emphasise the necessity to engineer SC mutants because introgression of *Sli* in the cultivated germplasm would result in linkage drag of undesirable *S. chacoense* traits such as long stolons or high tuber glycoalkaloid content. Because the *Sli* locus appears to be indigenous in cultivated potato germplasm, the use of genetically engineered plants can be circumvented.

Zhang et al. (2019a) proposed to introduce SC into *S. tuberosum* by crossing dihaploids from selected tetraploid varieties with M6 and other wild sources to introduce SC. Considering the widespread of the SC haplotypes among the tetraploid varieties, the one selected for dihaploids induction should include *Sli*. This suggests that the diploid SC gene pool can be expanded easily without the need for subsequent *Sli* introgression.

With this study, the challenges in diploid potato breeding will shift. The need for SC germplasm with agronomical value can be satisfied by screening genomes for specific *k*-mers or with the use of KASP markers. The next challenge is to develop material which remains highly fertile upon inbreeding. Male fertility appears as the next limitation to overcome and was already identified as an impediment to breeding in the early 20th century (Krantz 1924). Tetraploids cultivars with renowned spontaneous berry set should be excellent candidates for the induction of fertile dihaploids, but fertile and SC dihaploids such as G254 can be expected only if their tetraploid parents are *Sli*-bearing descendants from Rough Purple Chili.

## Electronic supplementary material

Below is the link to the electronic supplementary material.

**ESM 1:** Pedigrees of population IVP16-587 and IVP17-618. (PDF 25 kb)

**ESM 2:** Overview of DNA sequencing and *k*-mer data sets from IVP16-587 and IVP17-618 used for set operations. (XLSX 23 kb)

**ESM 3**: Frequency spectra of *k*-mers across all samples used in this study. (XLSX 120 kb)

**ESM 4:** Overview of *k*-mer data sets from the read archives used for set operations. (XLSX 26 kb)

**ESM 5:** Overview of phenotypic data for reproduction related traits in IVP16-587 and IVP17-618. (XLSX 32 kb)

**ESM 6:** Boundaries of the SC haplotype. IVG viewer was used to show map positions of SC specific *k*-mers on DM v4.03. (PDF 606 kb)

**ESM 7:** Heatmap displaying the density of SC specific *k*-mers on the distal end of chromosome *12* for all Hardigan et al. (2017) samples. (PDF 1016 kb)

**ESM 8:** Allele dosage estimation of *Sli* in diploids and tetraploids. (XLSX 56 kb)

**ESM 9:** Conversion of *k*-mers into KASP markers and validation of KASP marker predictions using a panel of diploid clones with known self-compatibility/fertility phenotype. (XLSX 42 kb)

**ESM 10:** Dendrogram of clones used in this study based on neighbour-joining clustering of Mash distance. Colour used is based on Fig. 5. (PDF 28 kb)
