# Supplementary material for: The origin and widespread occurrence of Sli-based self-compatibility in potato
Source: Theor Appl Genet. 2020 Jun 8;133(9):2713–28. doi: 10.1007/s00122-020-03627-8 (PMC7419354; doi:10.1007/s00122-020-03627-8)

Clot et al, The origin and widespread occurrence of Sli based self-compatibility in potato.

ESM1: Pedigrees of population IVP16-587 and IVP17-618. Spontaneous berry set has been observed in the clones highlighted in blue

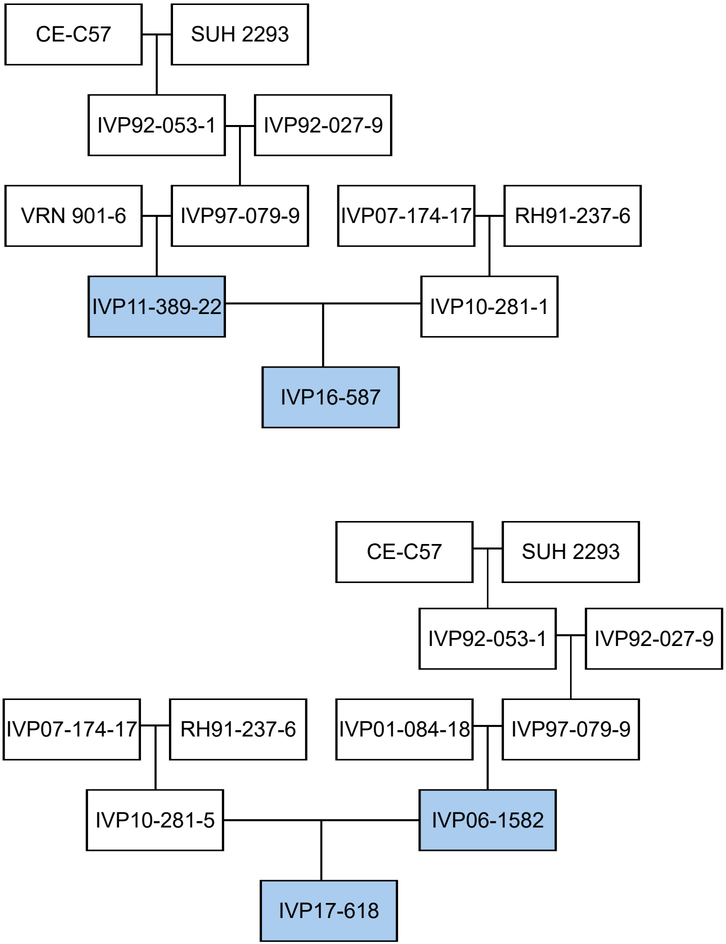

Supplement: Supplementary file 1 — ESM 1: Pedigrees of population IVP16-587 and IVP17-618. (PDF 25 kb) [file 122_2020_3627_MOESM1_ESM.pdf]
