# Supplementary material for: The origin and widespread occurrence of Sli-based self-compatibility in potato
Source: Theor Appl Genet. 2020 Jun 8;133(9):2713–28. doi: 10.1007/s00122-020-03627-8 (PMC7419354; doi:10.1007/s00122-020-03627-8)

Clot et al, The origin and widespread occurrence of Sli based self-compatibility in potato.
ESM6: Boundaries of the SC haplotype. IVG viewer was used to show map positions of SC specific k-mers on DM v4.03.

a) Intersection between k-mers in coupling with SC in IVP16-587 and IVP17-618

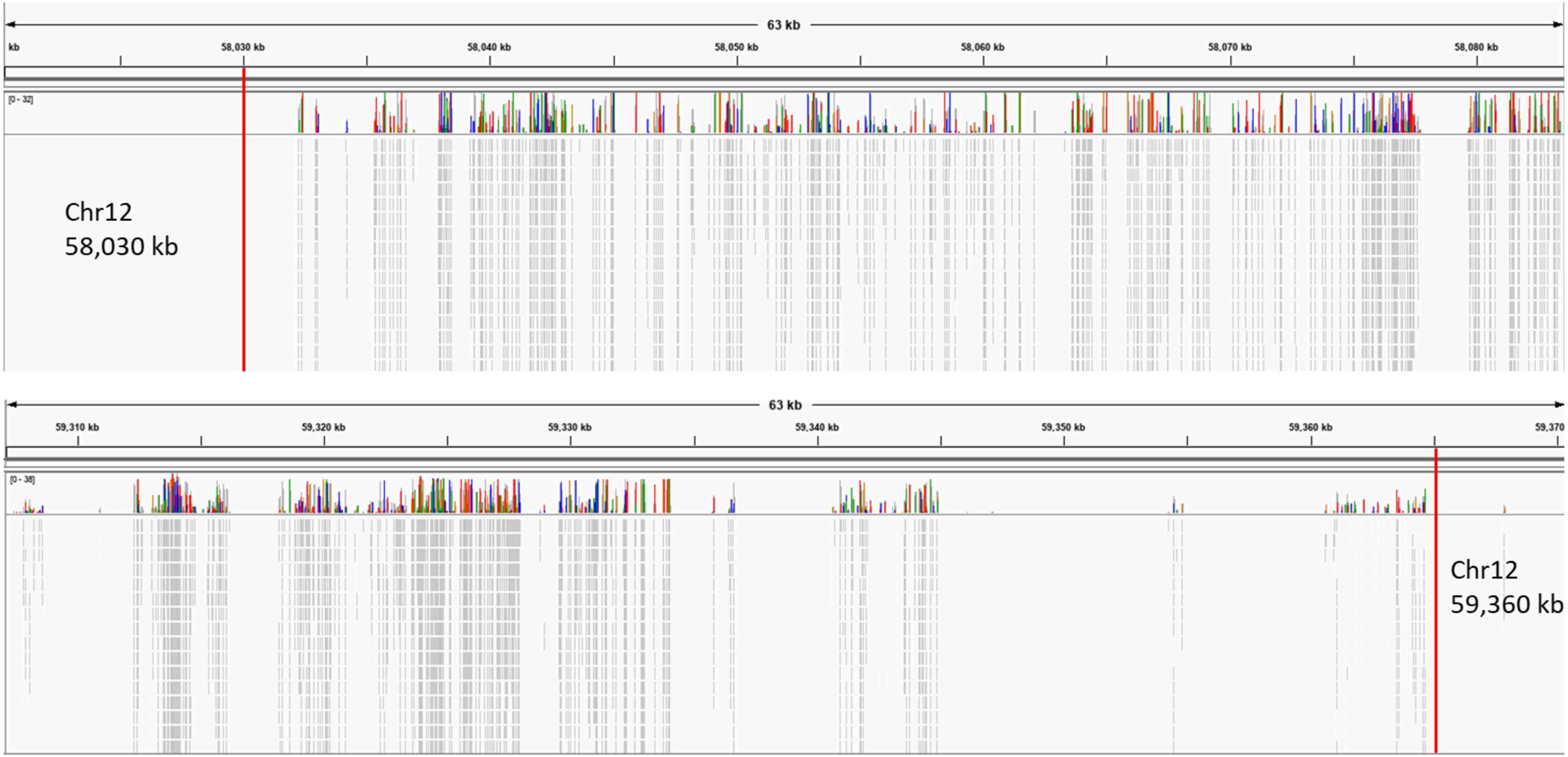

b) Intersection of shared k-mers in coupling phase with SC in IVP16-587 and IVP17-618 and M6 (*Sli* candidate region)

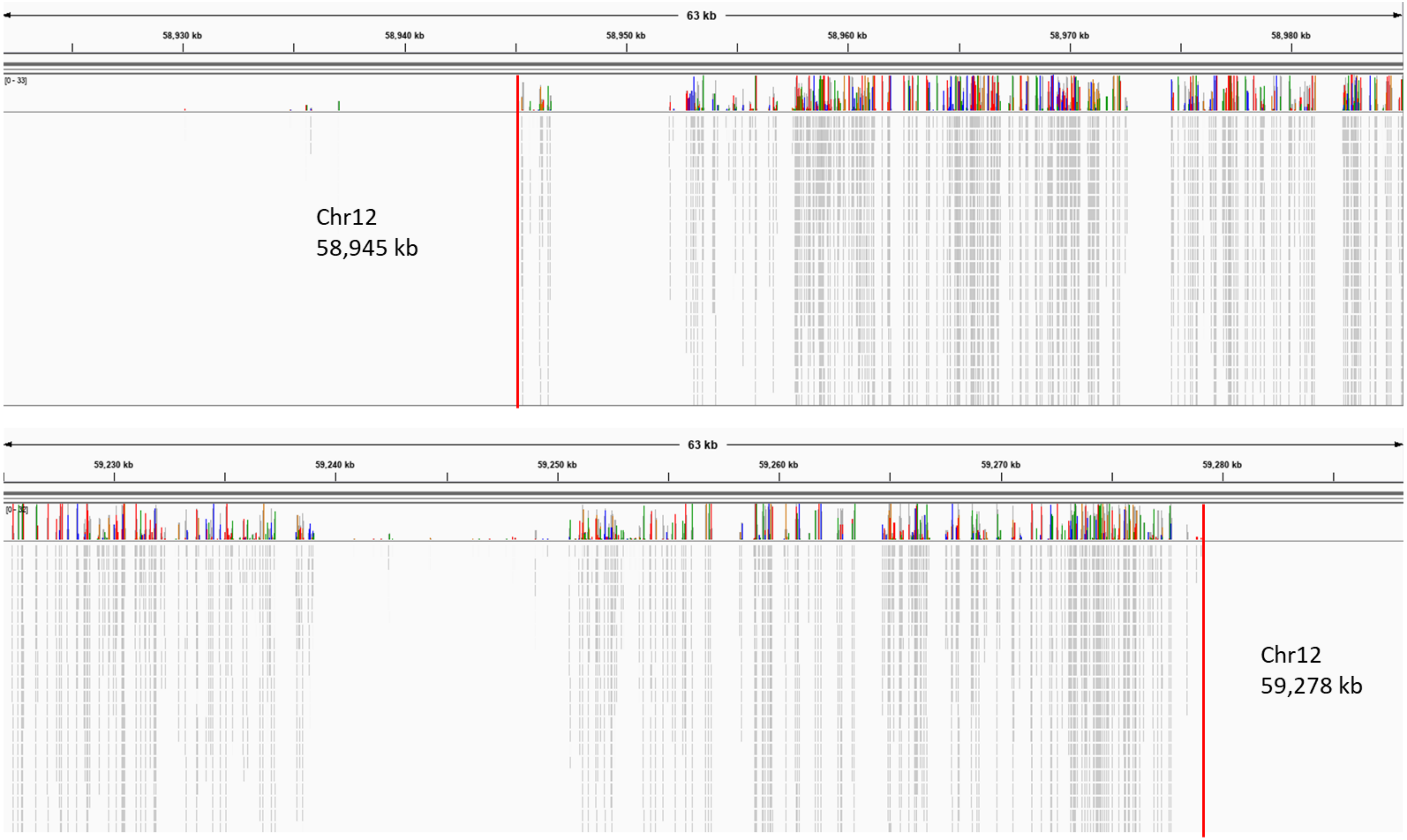

Supplement: Supplementary file 6 — ESM 6: Boundaries of the SC haplotype. IVG viewer was used to show map positions of SC specific k-mers on DM v4.03. (PDF 606 kb) [file 122_2020_3627_MOESM6_ESM.pdf]
