# Supplementary figures and images for: The origin and widespread occurrence of Sli-based self-compatibility in potato
Source: Theor Appl Genet. 2020 Jun 8;133(9):2713–28. doi: 10.1007/s00122-020-03627-8 (PMC7419354; doi:10.1007/s00122-020-03627-8)

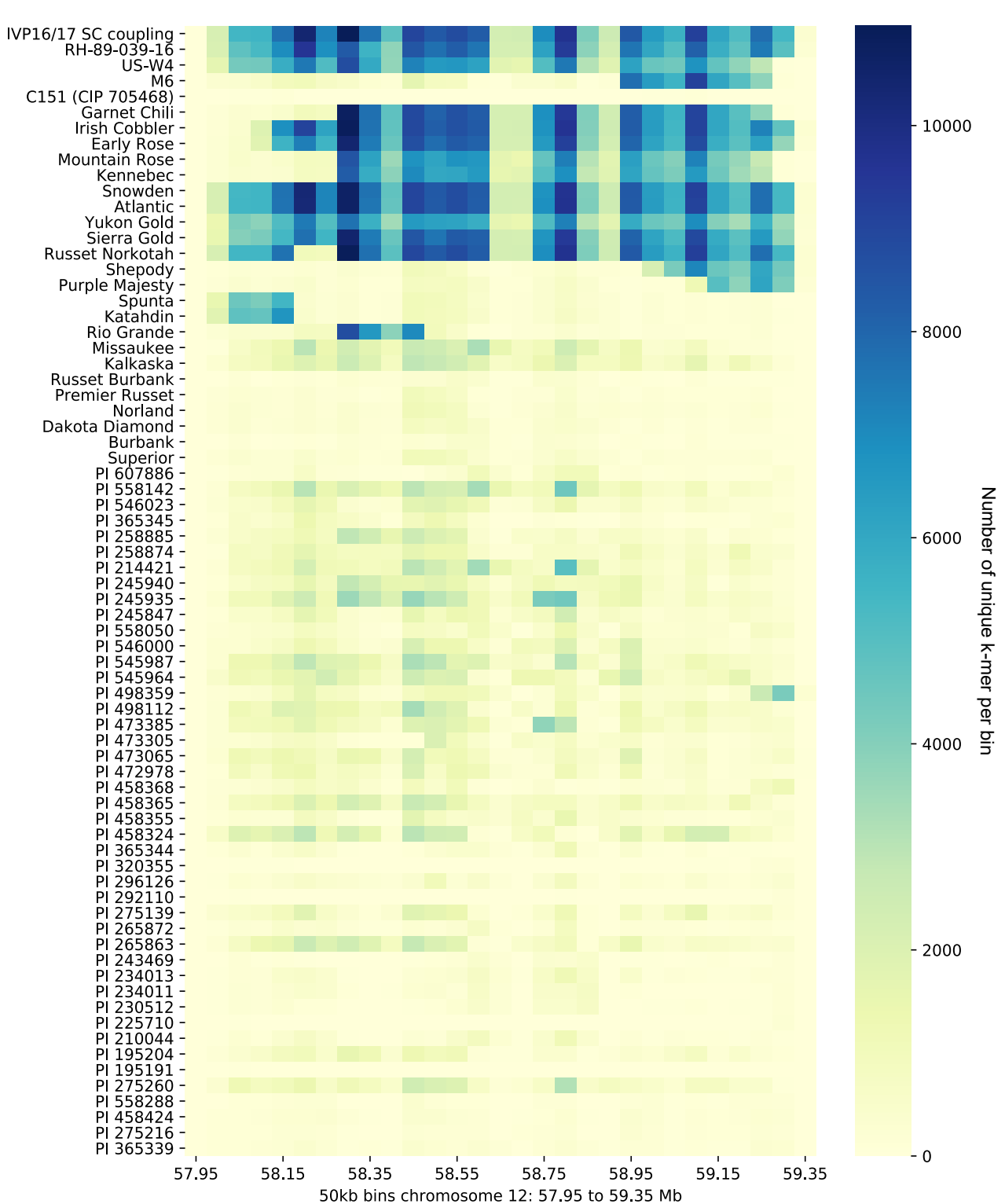

Supplement: Supplementary file 7 — ESM 7: Heatmap displaying the density of SC specific k-mers on the distal end of chromosome 12 for all Hardigan et al. (2017) samples. (PDF 1016 kb) [file 122_2020_3627_MOESM7_ESM.pdf]

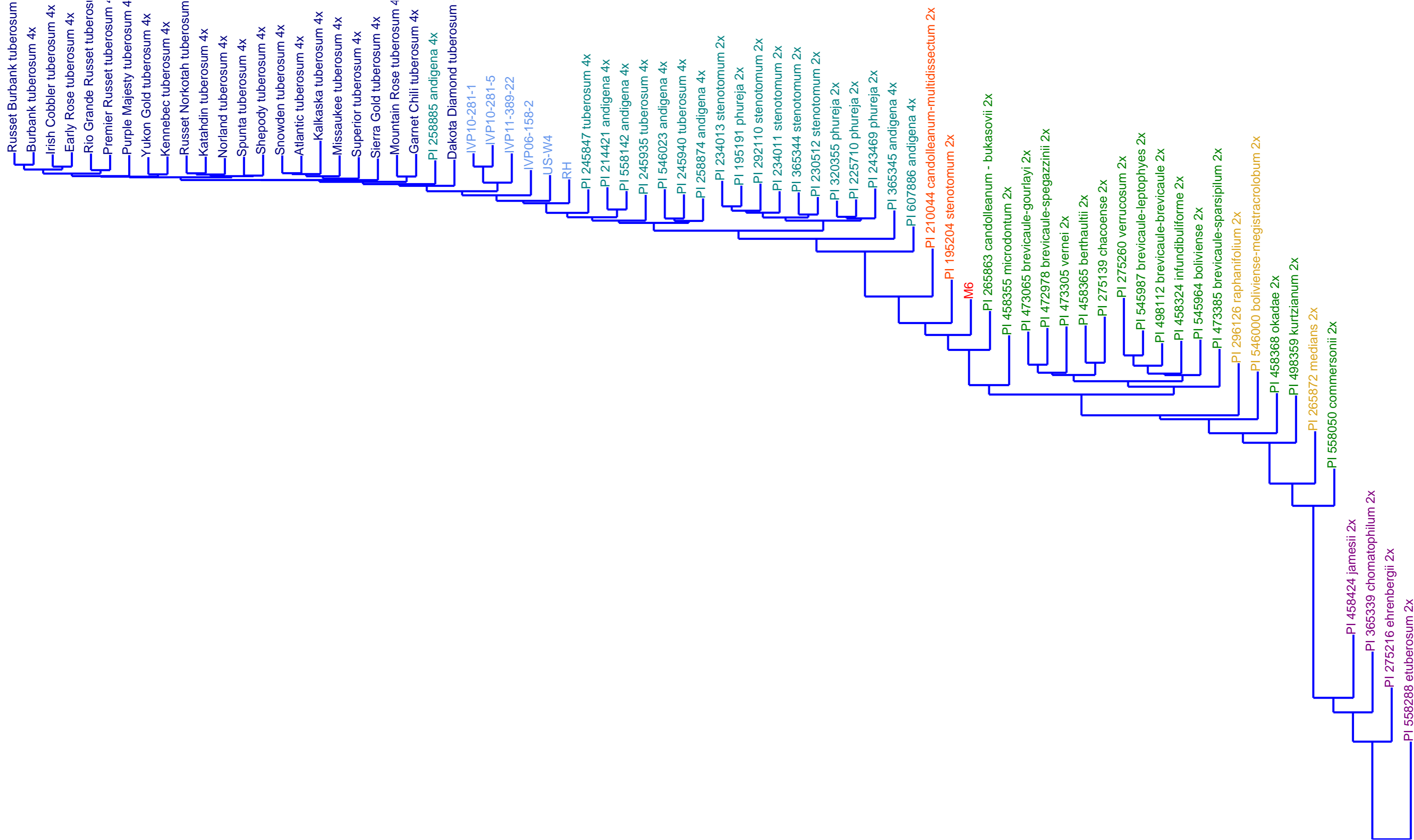

Supplement: Supplementary file 10 — ESM 10: Dendrogram of clones used in this study based on neighbour-joining clustering of Mash distance. Colour used is based on Fig. 5. (PDF 28 kb) [file 122_2020_3627_MOESM10_ESM.pdf]
